# Prof. D. W. Yandell

**Published:** 1867-10

**Authors:** 


### PROF. D. W. YANDELL.

We notice that Prof. Yandell, who fills the chair of "Principles and Practice of Medicine," in the Medical Department of the University of Louisville, was to deliver the introductory address before the students and faculty of that institution at the commencement of its regular session, on the 1st inst. We do not hesitate to say, although we have not seen the address, that it was an able one.

Prof. Yandell has no superior as a thinker and man of talents, of his age. His acquirements, varied, and of the first order—combining the useful with the elegant, show at once the crucible of thought, the grace of liturature, and felicity of style. His convictions led him into the Southern Army, at the very commencement of the war, although at a heavy sacrifice, and he stood by the cause, giving his earnest attention to the organization and efficiency of the Medical Department, until all was lost, and then returned to his native State, with his parole and oath of allegiance in his pocket, to resume and fulfill, like a man as he is, his duty to society, and all that is meant by allegiauce to the United States. We predict for him a future of great usefulness to the cause of humanity and of honor to himself and the profession, of which he is an ornament.

